# Imaging features of hyperacute intracerebral hemorrhage on multiple MRI sequences within 1 minute from onset during MRI examination: A case report

**DOI:** 10.1097/MD.0000000000033350

**Published:** 2023-03-31

**Authors:** Sang Bin Bae, Sanghyeon Kim

**Affiliations:** a Department of Radiology, Dong-A University College of Medicine, Seo-gu, Busan, Republic of Korea.

**Keywords:** hyperacute intracerebral hemorrhage, MRI, stroke

## Abstract

Acute stroke requires accurate imaging to ensure appropriate treatment decisions and favorable clinical outcomes. Computed tomography has long been used as an exclusive imaging technique to assess intracerebral hemorrhage, owing to its rapid scanning time and widespread availability. Several recent studies have reported the reliable detection of hyperacute hemorrhage using magnetic resonance imaging (MRI).

**Patient concerns:**

An 88-year-old woman with a history of hypertension presented with mild, acute dysarthria. The National Institutes of Health Stroke Scale score was 1.

**Diagnoses:**

Non contrast head computed tomography revealed the absence of acute cerebral hemorrhage. The patient underwent magnetic resonance, revealed hyperacute intracerebral hemorrhage within a few minutes of its occurrence on multiple MRI sequences.

**Interventions and outcomes:**

In this patient, hemorrhage developed during MRI for acute ischemic stroke. Hemorrhage was initially misdiagnosed, and inappropriate treatment severely affected the patient’s health.

**Lessons:**

Clinicians in the Department of Neurological Emergency should be familiar with imaging findings of hyperacute hemorrhage on multiple MRI sequences.

## 1. Introduction

Imaging plays a critical role in the evaluation and treatment of acute ischemic stroke. Because intravenous thrombolysis and endovascular therapy are contraindicated if intracerebral hemorrhage (ICH) is simultaneously detected, it is important to use imaging to rule out hemorrhage early.

Computed tomography (CT) has long been used as an exclusive imaging technique to assess ICH, owing to its rapid scanning time and widespread availability. Several recent studies have reported the reliable detection of hyperacute hemorrhage using magnetic resonance imaging (MRI).^[1]^ However, there are no reports showing various imaging features of hyperacute hemorrhage on multiple MRI sequences. Here, we report various imaging characteristics of a case of hyperacute ICH that developed during MRI for acute ischemic stroke. In the present case, the hemorrhage was initially misdiagnosed, resulting in inappropriate treatment that had devastating consequences on the patient’s health.

## 2. Case

**Figure 1.**
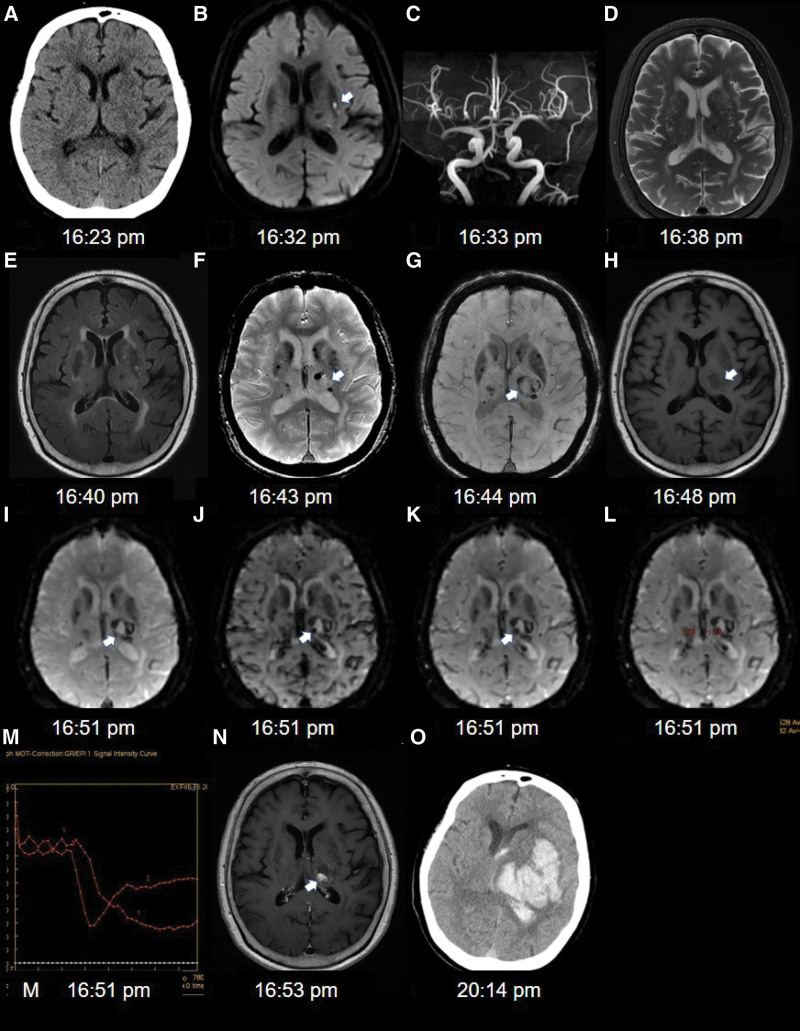
An 88-year-old female patient presented with acute mild dysarthria. (A) Non-contrast head CT shows no evidence of intracerebral hemorrhage, (B) DWI shows acute infarct in the left basal ganglia (arrow), (C) MRA shows a normal appearance of the intracranial vessels, (D and E) T2WI and FLAIR image show no definite hyperintensity in the left thalamus, (F) GRE image shows multiple microbleeds in both the basal ganglia and thalamus. Mild hyperintense signal change is observed in the left thalamus, which is absent in previous T2WI and FLAIR image (arrow), (G) SWI shows a dark signal rim around the periphery of the hyperacute hematoma in the left thalamus (arrow), (H) T1WI demonstrates a lesion of low signal intensity in the same area as GRE image (arrow), (I–K) In T2*-weighted images from DSC-MRI, an area of dark signal intensity disperses and spreads out within the hematoma (arrow). The time interval between images is 20 seconds, (L and M) In the region of interest (ROI 1), the time-intensity curve shows a persistent signal decrease compared with that in the contralateral normal region of interest (ROI 2), (N): Post-contrast T1WI shows a collection of extravasated contrast along the margin of the hematoma (arrow), and (O) Follow up non-contrast head CT at 3 hours after the MRI demonstrates marked enlargement of the hemorrhage. CT = computed tomography, DSC = dynamic susceptibility contrast, DWI = diffusion-weighted imaging, FLAIR = fluid-attenuated inversion recovery, GRE = gradient recall echo, MRA = magnetic resonance angiography, MRI = magnetic resonance imaging, SWI = susceptibility-weighted image, T1WI = T1-weighted imaging, T2WI = T2-weighted imaging.

An 88-year-old woman with a history of hypertension presented with mild, acute dysarthria. The National Institutes of Health Stroke Scale score was 1. Non-contrast head CT revealed the absence of an acute cerebral hemorrhage (Fig. 1A). The patient underwent a magnetic resonance (MR) examination using a 3.0 T MR system (Milwaukee, WI) following the study protocols, including diffusion-weighted imaging (DWI), MR angiography (MRA), T2-weighted imaging (T2WI), fluid-attenuated inversion recovery (FLAIR), T2*-weighted gradient recall echo (GRE) sequence, T1-weighted imaging (T1WI), susceptibility-weighted imaging (SWI), dynamic susceptibility contrast (DSC), perfusion-weighted imaging (PWI), and post-contrast T1WI. DWI demonstrated restricted diffusion in the basal ganglia (Fig. 1B). MRA showed a normal appearance of the intracranial vessels (Fig. 1C). T2WI and FLAIR images revealed no hyperintensity in the thalamus (Fig. 1D and E). GRE images showed multiple microbleeds in the brain (Fig. 1F). After analyzing only DWI, MRA, T2WI, FLAIR, and GRE images, the clinician cautioned against the potential risk of hemorrhage due to cerebral microbleeds, and prescribed aspirin for acute ischemic stroke. The remaining MRI sequences were performed according to the protocol without a clinician. The patient’s dysarthria worsened immediately after MRI examination, and the patient deteriorated into a stupor. Follow up non-contrast head CT performed 3 hours later showed a large hyperdense hematoma in the left thalamus and basal ganglia (Fig. 1O).

A thorough MRI analysis subsequently demonstrated a subtle increase in signal intensity in the thalamus on GRE images, which was otherwise absent in b0, T2WI, and FLAIR images. This hyperintensity was interpreted as hyperacute hemorrhage. SWI revealed a dark rim surrounding the hematoma (Fig. 1G). T1WI revealed hypo intensity in the left thalamus (Fig. 1H). DSC PWI revealed a persistent size increase and signal decrease in the dark signal intensity over time (Fig. 1H–M). Post-contrast T1WI revealed multiple nodular or rim enhancements along the hematoma margins (Fig. 1N). The MRI findings indicated active contrast extravasation.

This case report was approved by our Institutional Review Board and the requirement for written informed consent was waived (DAUHIRB-22-253).

## 3. Discussion

Our case reports the various imaging characteristics of hyperacute ICH within a few minutes of its occurrence on multiple MRI sequences. Hyperacute hemorrhage appears hyperintense on T2WI, FLAIR, and GRE images, owing to its protein-rich water content.^[2]^ In our case, T2WI and FLAIR images were normal, but GRE images showed hyperintensity, indicating that cerebral hemorrhage occurred within the time interval between the FLAIR and GRE images. A peripheral hypointense rim on GRE images usually appears within 1 hour of hematoma progression and is attributed to the early formation of deoxyhemoglobin, which possesses magnetic susceptibility.^[3]^ Modern SWI is more sensitive to microhemorrhage detection than GRE.^[1]^ We confirmed the presence of this hypointense rim on SWI.

Hematoma expansion in ICH is considered an independent predictor of early neurological deterioration, poor functional outcome, and mortality. Contrast extravasation into the hematoma cavity is associated with an active hemorrhage and subsequent hematoma expansion.^[4–6]^ In our case, post-contrast T1WI imaging demonstrated an MRI spot sign with multiple small nodular enhancements along the margins of the hematoma. The accumulation of contrast material in ruptured vessels results in an increased longitudinal relaxation rate and consequently, increased signal intensity on contrast-enhanced T1WI. This contrast enhancement represents a collection of extravasated gadolinium contrast and must not be confused with contrast enhancement owing to a tumor or vascular malformation. DSC PWI showed a dispersed increase in the size of dark spots. The dark spots continued to spread throughout the hematoma. The lesion was consistently located with a focus on contrast enhancement observed on contrast-enhanced T1WI. Furthermore, the signal drop in the time-signal intensity curve was prolonged, with no return to baseline. These MRI findings support the contrast leakage observed on contrast-enhanced T1WI as an ongoing bleeding.^[7,8]^

Cerebral microbleeds have previously been recognized as a risk factor for ICH.^[9]^ Cerebral amyloid angiopathy and hypertension are the main causes of cerebral microbleeds. Hypertension-related microbleeds typically involve the basal ganglia, thalamus, and the infratentorial brain.^[10]^ Our patient had a history of chronic hypertension and demonstrated multiple susceptibility foci in the basal ganglia, thalamus, brain stem, and cerebellum, suggesting hypertension-related microbleeds as the underlying etiology of intracerebral hemorrhage.

## 4. Conclusion

Misdiagnosis may occur, in part, because of the immense time pressure on attending clinicians in the Department of Neurological Emergency. However, the accurate interpretation of imaging findings can affect treatment decisions for stroke, thus improving clinical outcomes. Clinicians should be familiar with various MR features of hyperacute hemorrhage on multiple sequences.

## Author contributions

**Conceptualization:** Sanghyeon Kim.

**Data curation:** Sanghyeon Kim.

**Writing – original draft:** Sang Bin Bae, Sanghyeon Kim.

**Writing – review & editing:** Sanghyeon Kim.
